# Exploring Employment Trend and Relationship With Individual and Family-Related Factors in 65 or Older Koreans

**DOI:** 10.1093/geroni/igaa057.212

**Published:** 2020-12-16

**Authors:** Yeonji Ryou, Ryou Yeonji

**Affiliations:** Iowa State University, Ames, Iowa, United States

## Abstract

The purpose of this study is to identify the trend of the employment status in 65 years or older adults who reside in South Korea and to explore the relationship between the status of employment and individual and family-related factors. This study utilized 10-year and 6-wave secondary data from the Korean Longitudinal Study of Ageing (KLoSA). The original panel sample is a random sample of 10,254 adults who are 45 or older, but for the aim of this study, the participants younger than 65 years were excluded. The number of samples in each wave is different, ranging from 4,013 to 4,335 due to the death of the participant, the rejection of additional interviews, and the refreshment participant collected in Wave 5. The findings indicate that the absolute employment of the people aged 65 or older and the proportion of working people among those have increased over the past decade. In this study, it is also found that there is a close relationship between employment status and individual factors such as gender, educational background, health condition, region, etc. Moreover, the results suggest that there are various facets of the relationship between employment status and family-related factors including whether living with children, the number of the member whom I help with daily activities, the total amount of financial support from/to children/parents/other family or whether participating social activities, etc. The implications of the need for employing the older population and the consideration family-related factors in the policy-making process in Korea are discussed.

